# Supplementary figures and images for: The Exon Junction Complex and intron removal prevent re-splicing of mRNA
Source: PLoS Genet. 2021 May 25;17(5):e1009563. doi: 10.1371/journal.pgen.1009563 (PMC8184009; doi:10.1371/journal.pgen.1009563)

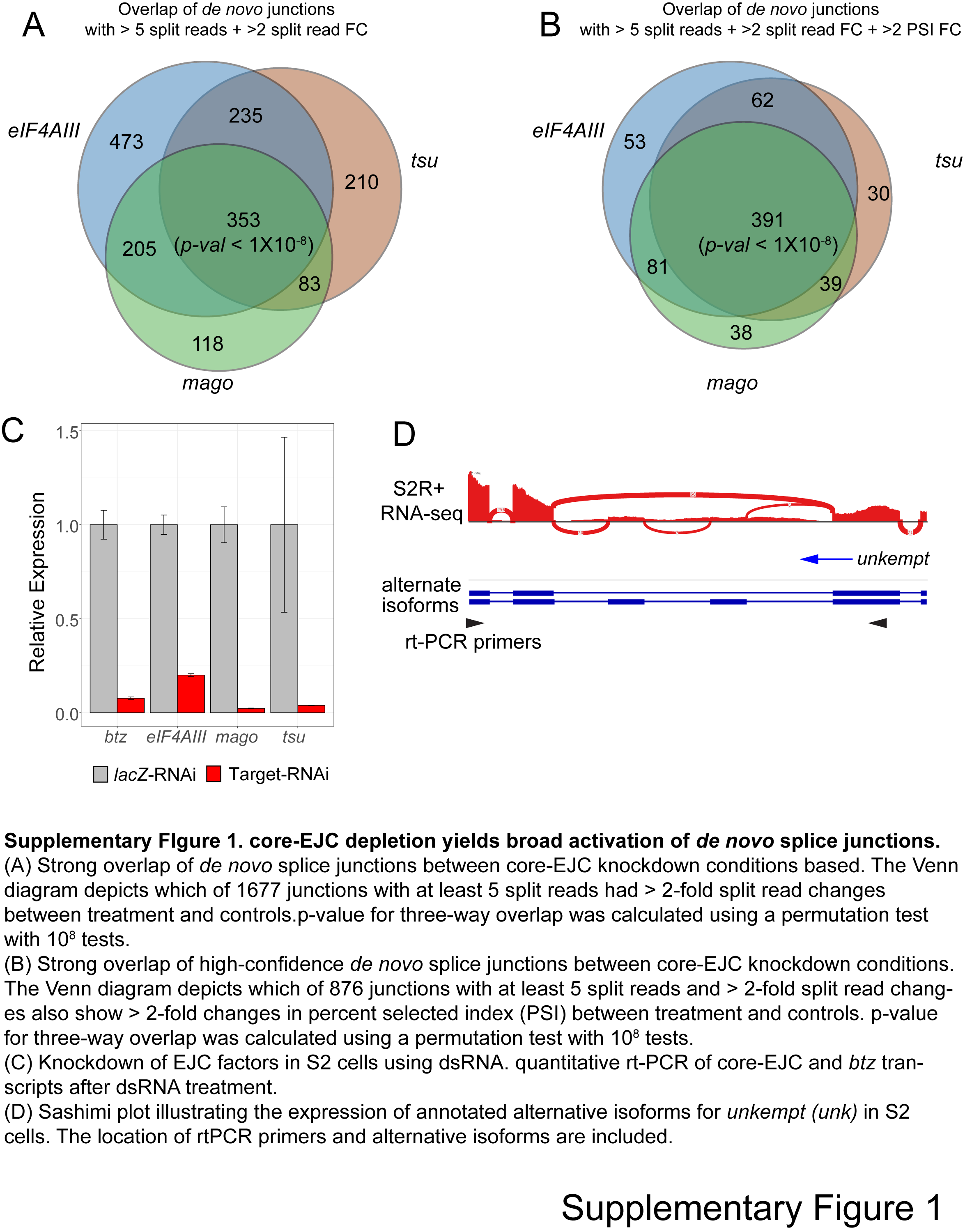

Supplement: S1 Fig — (A) Strong overlap of de novo splice junctions between core-EJC knockdown conditions. The Venn diagram depicts which of 1677 junctions with at least 5 split reads had > 2-fold split read changes between treatment and controls. p-value for three-way overlap was calculated using a permutation test with 10^8 tests. (B) Strong overlap of high-confidence de novo splice junctions between core-EJC knockdown conditions. The Venn diagram depicts which of 876 junctions with at least 5 split reads and > 2-fold split read changes also show > 2-fold changes in percent selected index (PSI) between treatment and controls. p-value for three-way overlap was calculated using a permutation test with 10^8 tests. (C) Knockdown of EJC factors in S2 cells using dsRNA. quantitative rt-PCR of core-EJC and btz transcripts after dsRNA treatment. (D) Sashimi plot illustrating the expression of annotated alternative isoforms for unkempt (unk) in S2 cells. The location of rtPCR primers and alternative isoforms are included. (TIF) [file pgen.1009563.s001.tif]

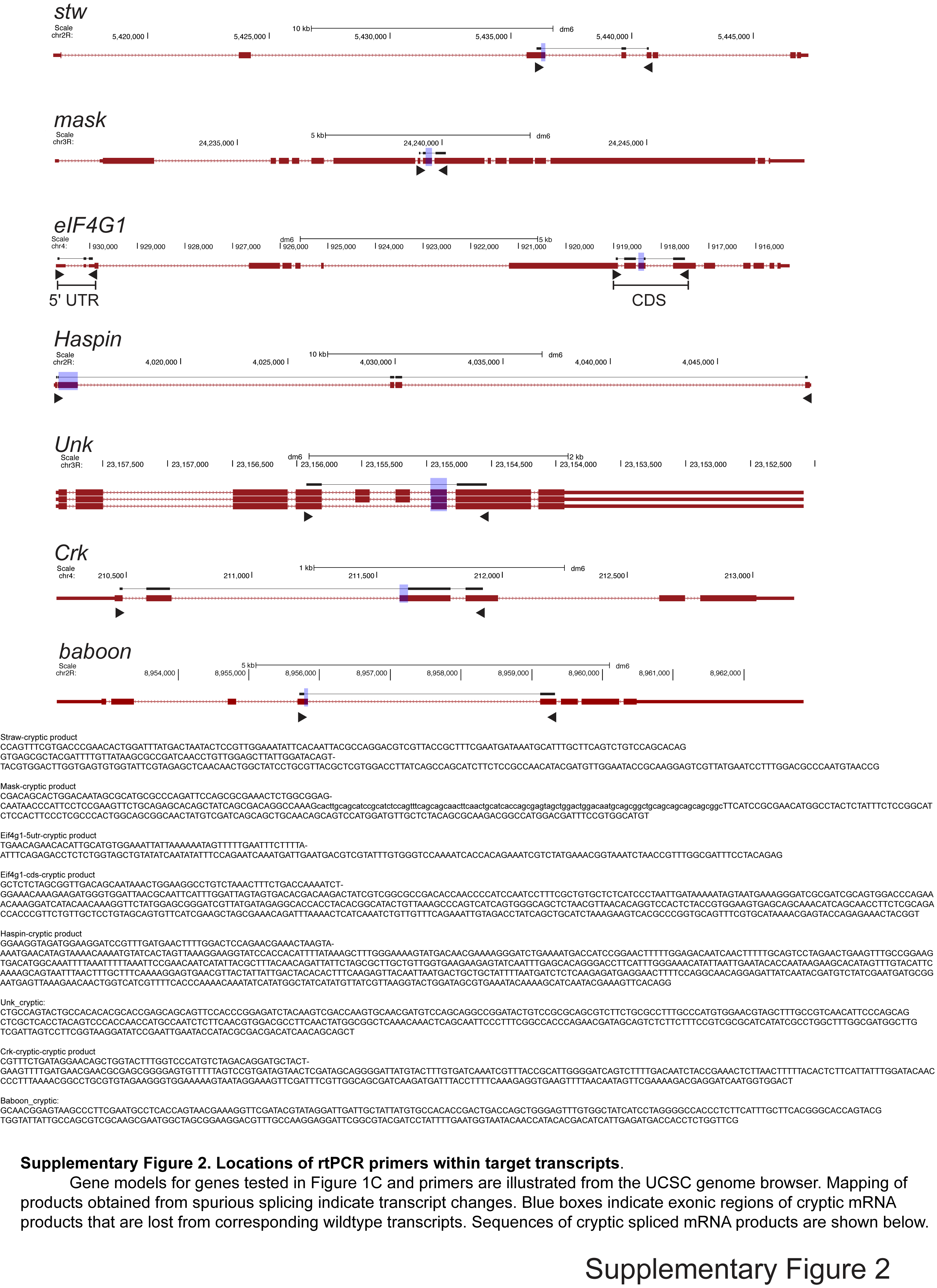

Supplement: S2 Fig — Gene models for genes tested in Fig 1C and primers are illustrated from the UCSC genome browser. Mapping of products obtained from spurious splicing indicate transcript changes. (TIF) [file pgen.1009563.s002.tif]

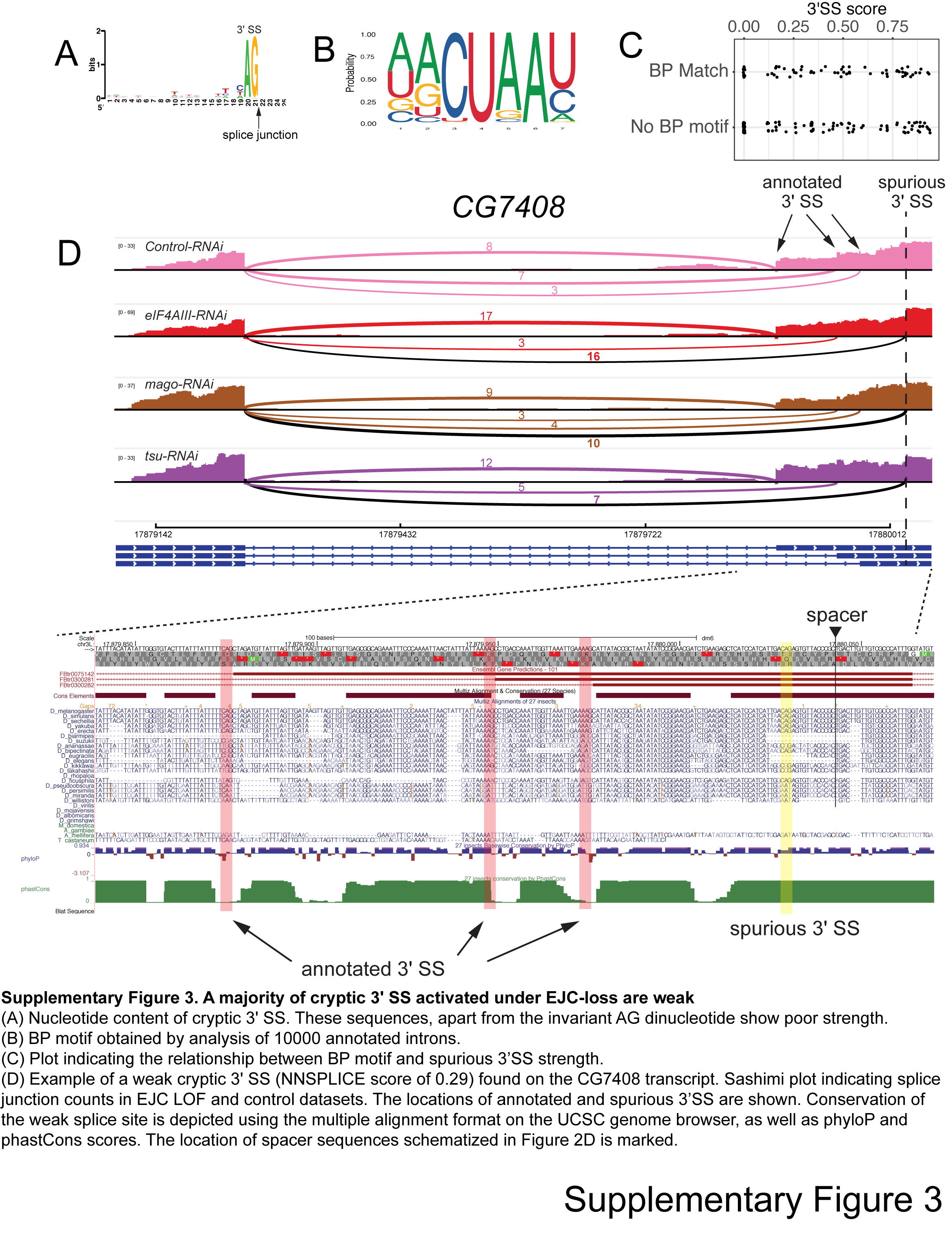

Supplement: S3 Fig — (A) Nucleotide content of cryptic 3’ SS. These sequences, apart from the invariant AG dinucleotide show poor strength. (B) BP motif obtained by analysis of 10000 annotated introns. (C) Plot indicating the relationship between BP motif and spurious 3’ SS strength. (D) Example of a weak cryptic 3’ SS (NNSPLICE score of 0.29) found on the CG7408 transcript. Sashimi plot indicating splice junction counts in EJC LOF and control datasets. The locations of annotated and spurious 3’ SS are shown. Conservation of the weak splice site is depicted using the multiple alignment format on the UCSC genome browser, as well as phyloP and phastCons scores. The location of spacer sequences schematized in Fig 2D is marked. (TIF) [file pgen.1009563.s003.tif]

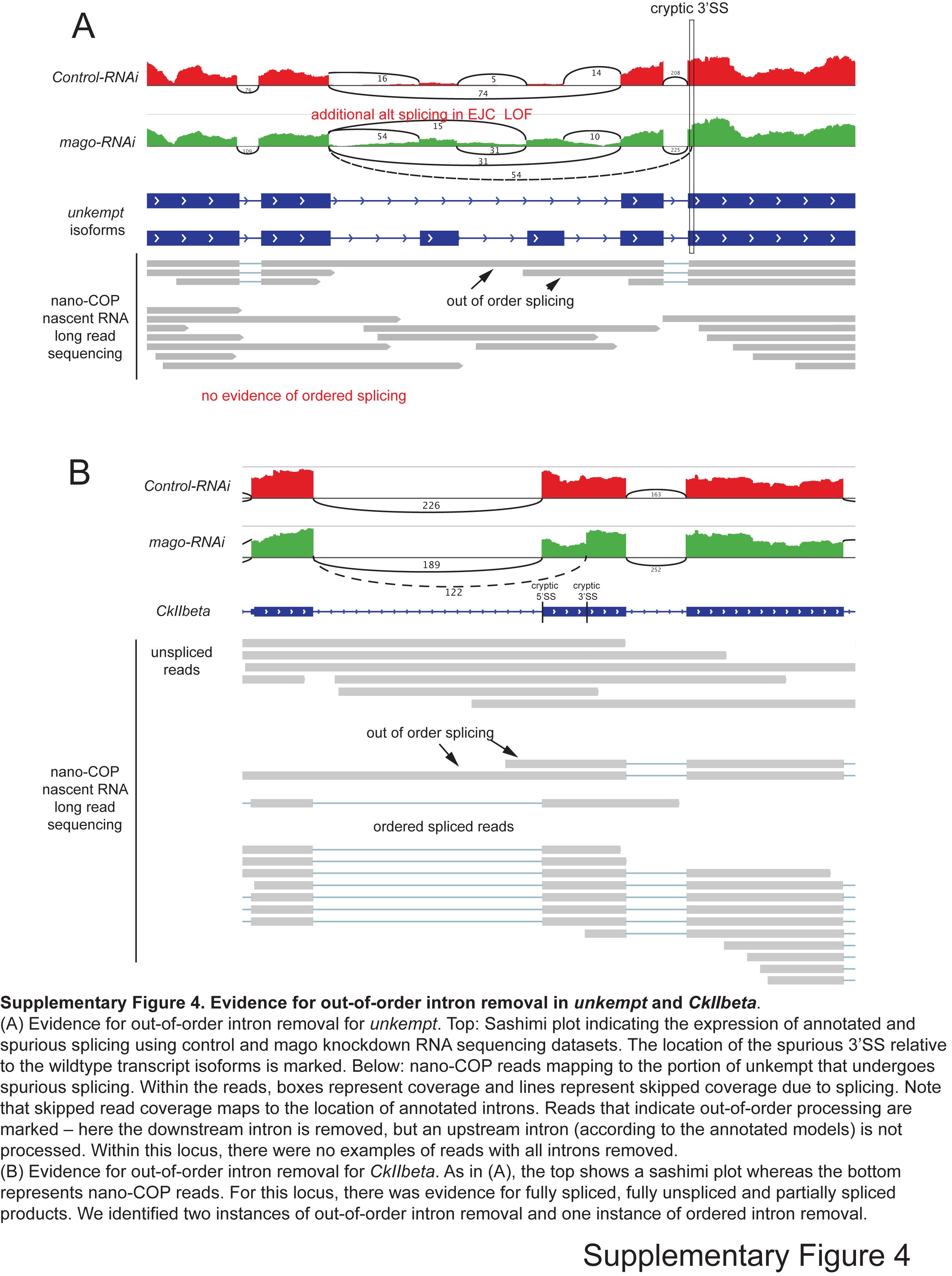

Supplement: S4 Fig — (A) Evidence for out-of-order intron removal for unkempt. Top: Sashimi plot indicating the expression of annotated and spurious splicing using control and mago knockdown RNA sequencing datasets. The location of the spurious 3’ SS relative to the wildtype transcript isoforms is marked. Below: nano-COP reads mapping to the portion of unkempt that undergoes spurious splicing. Within the reads, boxes represent coverage and lines represent skipped coverage due to splicing. Note that skipped read coverage maps to the location of annotated introns. Reads that indicate out-of-order processing are marked–here the downstream intron is removed, but an upstream intron (according to the annotated models) is not processed. Within this locus, there were no examples of reads with all introns removed. (B) Evidence for out-of-order intron removal for CkIIβ. As in (A), the top shows a sashimi plot whereas the bottom represents nano-COP reads. For this locus, there was evidence for fully spliced, fully unspliced and partially spliced products. We identified two instances of out-of-order intron removal and one instance of ordered intron removal. (TIF) [file pgen.1009563.s004.tif]

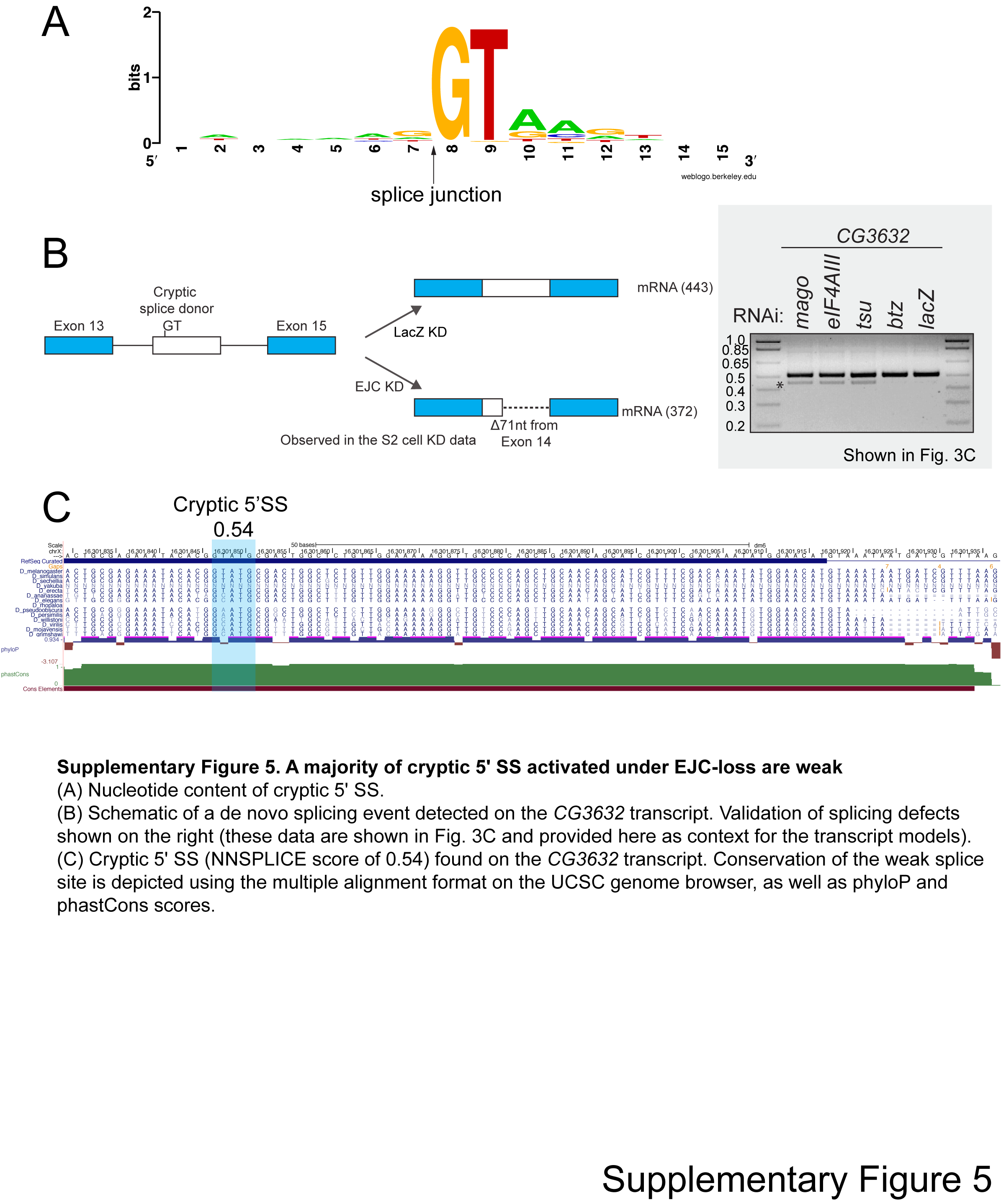

Supplement: S5 Fig — (A) Nucleotide content of cryptic 5’ SS. (B) Schematic of a de novo splicing event detected on the CG3632 transcript. Validation of splicing defects shown on the right. (C) Cryptic 5’ SS (NNSPLICE score of 0.54) found on the CG3632 transcript. Conservation of the weak splice site is depicted using the multiple alignment format on the UCSC genome browser, as well as phyloP and phastCons scores. (TIF) [file pgen.1009563.s005.tif]

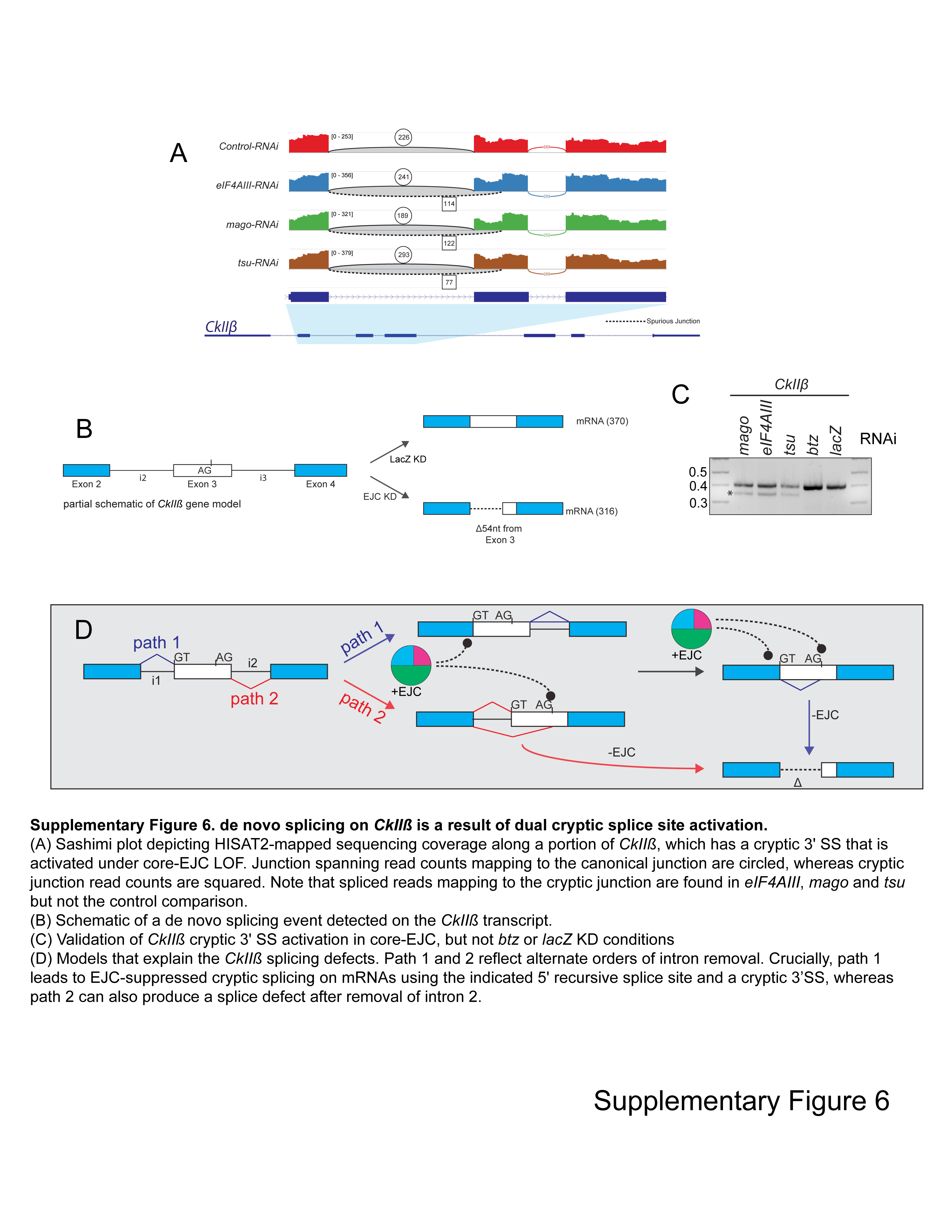

Supplement: S6 Fig — (A) Sashimi plot depicting HISAT2-mapped sequencing coverage along a portion of CkIIβ, which has a cryptic 3’ SS that is activated under core-EJC LOF. Junction spanning read counts mapping to the canonical junction are circled, whereas cryptic junction read counts are squared. Note that spliced reads mapping to the cryptic junction are found in eIF4AIII, mago and tsu but not the control comparison. (B) Schematic of a de novo splicing event detected on the CkIIβ transcript. (C) Validation of CkIIβ cryptic 3’ SS activation in core-EJC, but not btz or lacZ KD conditions. (D) Models that explain the CkIIβ splicing defects. Path 1 and 2 reflect alternate orders of intron removal. Crucially, path 1 leads to EJC-suppressed cryptic splicing on mRNAs using the indicated 5’ recursive splice site and a cryptic 3’ SS, whereas path 2 can also produce a splice defect after removal of intron 2. (TIF) [file pgen.1009563.s006.tif]

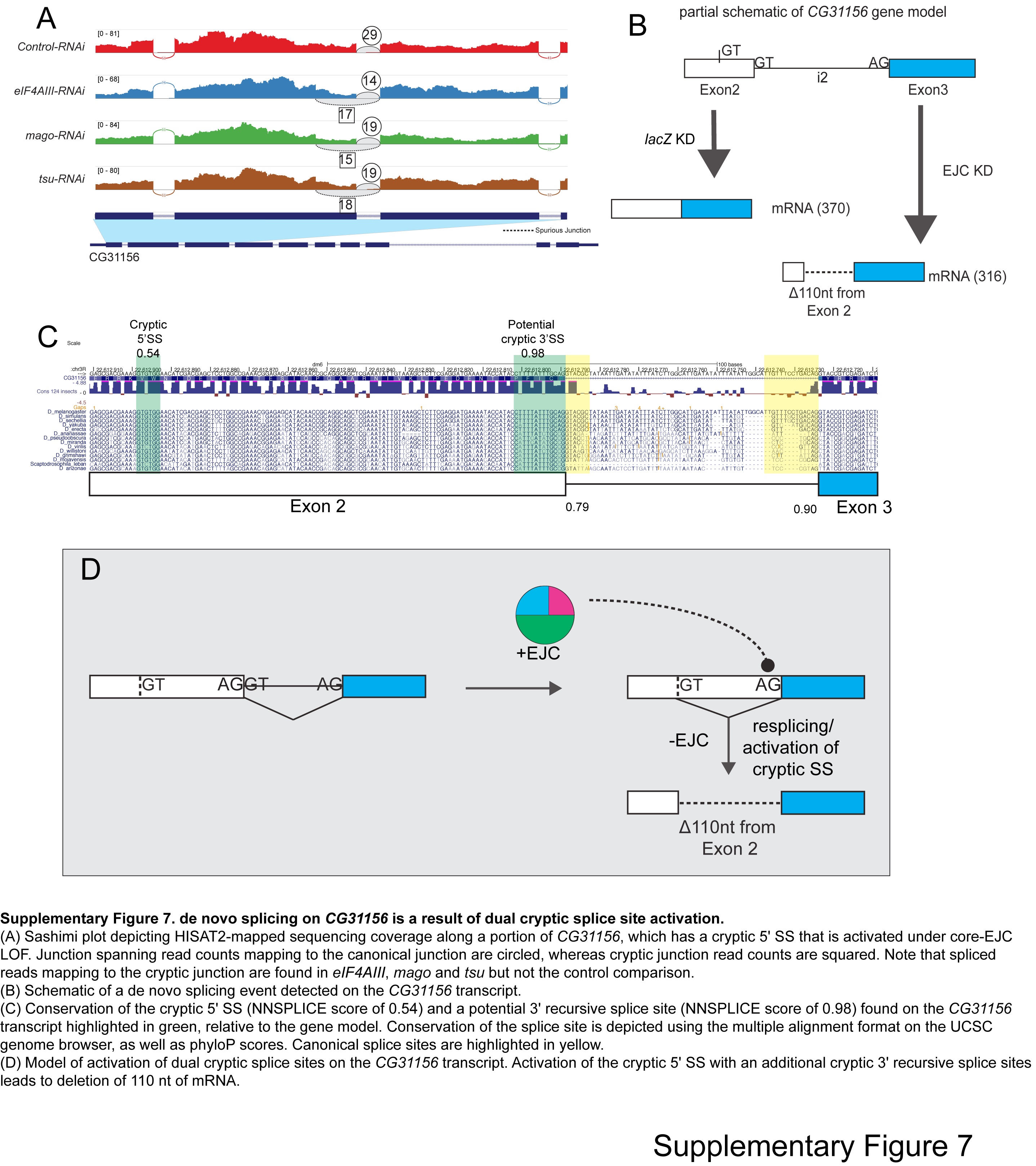

Supplement: S7 Fig — (A) Sashimi plot depicting HISAT2-mapped sequencing coverage along a portion of CG31156, which has a cryptic 5’ SS that is activated under core-EJC LOF. Junction spanning read counts mapping to the canonical junction are circled, whereas cryptic junction read counts are squared. Note that spliced reads mapping to the cryptic junction are found in eIF4AIII, mago and tsu but not the control comparison. (B) Schematic of a de novo splicing event detected on the CG31156 transcript. (C) Conservation of the cryptic 5’ SS (NNSPLICE score of 0.54) and a potential 3’ recursive splice site (NNSPLICE score of 0.98) found on the CG31156 transcript highlighted in green, relative to the gene model. Conservation of the splice site is depicted using the multiple alignment format on the UCSC genome browser, as well as phyloP scores. Canonical splice sites are highlighted in yellow. (D) Model of activation of dual cryptic splice sites on the CG31156 transcript. Activation of the cryptic 5’ SS with an additional cryptic 3’ recursive splice sites leads to deletion of 110 nt of mRNA. (TIF) [file pgen.1009563.s007.tif]

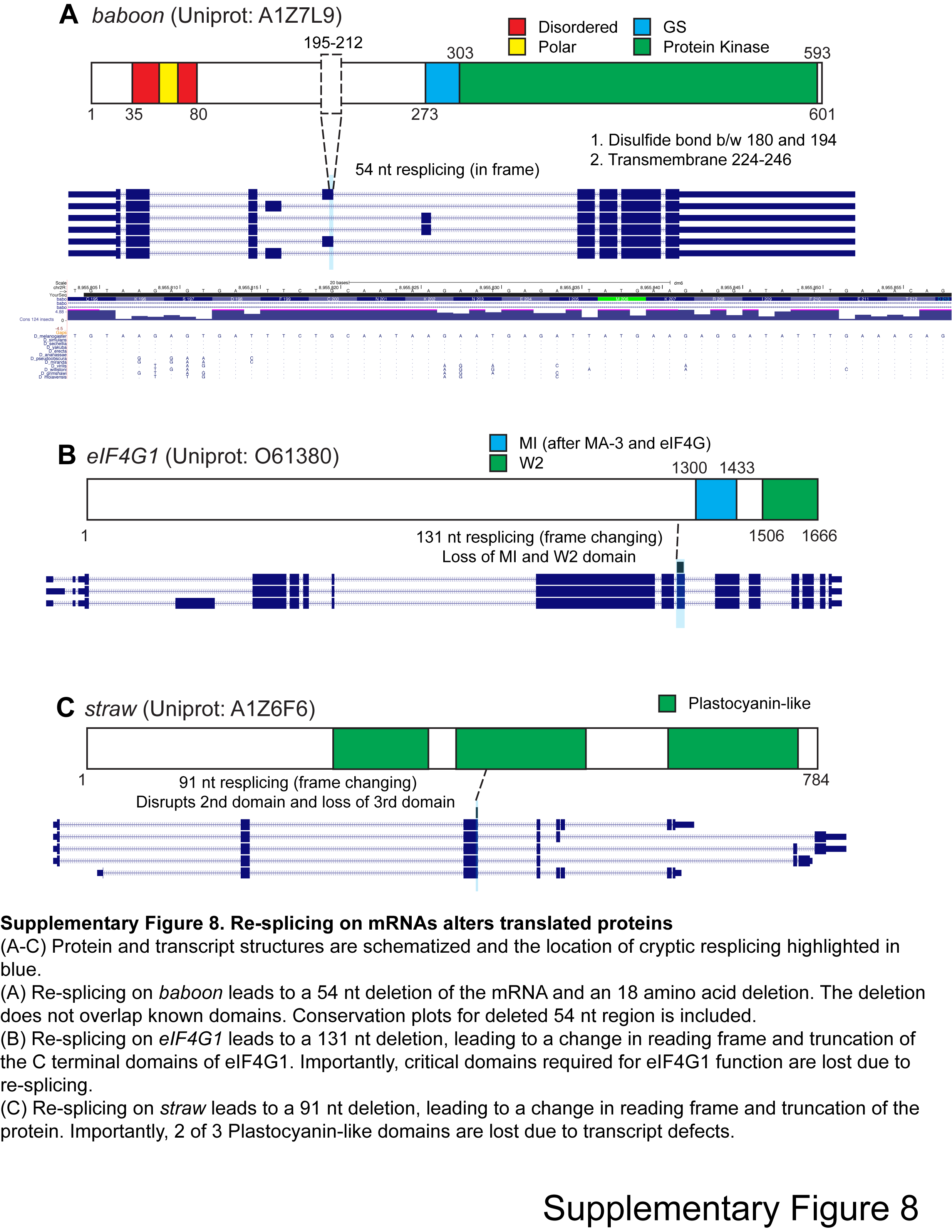

Supplement: S8 Fig — (A-C) Protein and transcript structures are schematized and the location of cryptic resplicing highlighted in blue. (A) Re-splicing on baboon leads to a 54 nt deletion of the mRNA and an 18 amino acid deletion. The deletion does not overlap known domains. Conservation plots for deleted 54 nt region is included. (B) Re-splicing on eIF4G1 leads to a 131 nt deletion, leading to a change in reading frame and truncation of the C terminal domains of eIF4G1. Importantly, critical domains required for eIF4G1 function are lost due to re-splicing. (C) Re-splicing on straw leads to a 91 nt deletion, leading to a change in reading frame and truncation of the protein. Importantly, 2 of 3 Plastocyanin-like domains are lost due to transcript defects. (TIF) [file pgen.1009563.s008.tif]
