# Supplementary material for: The Exon Junction Complex and intron removal prevent re-splicing of mRNA
Source: PLoS Genet. 2021 May 25;17(5):e1009563. doi: 10.1371/journal.pgen.1009563 (PMC8184009; doi:10.1371/journal.pgen.1009563)
Supplement: Attachment — Submitted filename: 2021_0225_PlosGen_EJC_ref_responses.pdf [file pgen.1009563.s012.pdf]

Reviewer's Responses to Questions
Comments to the Authors:
Please note here if the review is uploaded as an attachment.
Reviewer #1: The authors have reanalyzed RNA-seq data, previously published by Akhtar et al. 2019, for EJC knockdowns in Drosophila cells. They follow with RT-PCR of specific transcripts and from cells transfected with reporter constructs to provide experimental validation. Overall, they conclude that loss of EJC deposition results in activation of cryptic splice sites, as was found in human cells (Gehring, Mol. Cell 2018). The issue of the extent to which the EJC influences splicing is an interesting one, and I value such data from more than one model system. However, I am not convinced that the data in the current manuscript are sufficiently compelling on their own for many of the conclusions drawn. Rather, they are consistent with an interpretation already made in the human system.
Overall, the writing needs to be more precise, the presentation of the data more complete, and the description of what can and cannot be concluded from the data more accurate.

We thank the referee for their feedback. We tried to frame the relevant concepts and background findings in the introduction, although as mentioned, perhaps we were not as clear in laying these out as we could be. We value their comments as we certainly wish to convey these accurately and fully. However, we wish to point out that the finding that Drosophila shows regulatory features that "*are consistent with an interpretation already made in the human system*" is not merely a "me-too" set of findings. This is because a history of studies on EJC and splicing showed the fly EJC promotes splicing of various genes, rather than as we show, inhibits splicing as was recently shown in mammals. These "broadly consistent" conclusions about transcriptome control can only have come about from a comprehensive de novo re-annotation of Drosophila spliced junctions as we have done. Without this, the view in the literature is that the EJC is quite divergently employed in invertebrates and mammals, which itself could be consistent with other non-conserved, well-known usages of the EJC (eg, in nonsense mediated decay). We believe the revision has been improved by the referee comments and thank them for their careful attention, and hope they will find support for the current manuscript.

Specific Comments:
1 -p.2. I do not understand how the authors can justify saying “Unexpectedly, we discover the EJC inhibits scores of regenerated 5' and 3' recursive splice sites on segments that have already undergone splicing”, when this is exactly one of the findings of the Gehring Mol Cell paper.

We appreciate the reviewer’s concern and will reword our statements. However, we wish to emphasize key accomplishments of our manuscript in relation to the EJC’s role in suppressing re-splicing and the recursive splicing at large. To us, and the fly community, we believe it is appropriate to call these unexpected, because Drosophila is one of the most carefully and fully annotated transcriptomes. We have long participated in these studies as a core member of the modENCODE project and were responsible for several substantial updates to the fly annotation over the years. Yet, by focusing exclusively on unannotated junctions, we identified 573 genuine splicing events that arise in mutant conditions. This is not a minor revision to the transcriptome.

Furthermore, two important considerations motivate our statements regarding the importance and unexpected nature of this study: first, some functions of the EJC

described in the literature are divergent between mammals and invertebrates. For example, the mammalian EJC is critically involved in NMD, but not in invertebrates. Therefore, it was quite a surprise to us, that suppression of cryptic splice sites would be a key ancestral property. Second, while it is true the Gehring study demonstrated use of a recursive splice site that occurs at the 5' end of an exon (and ~6000 such examples were shared in the Blazguez 2018 Mol Cell), no one to date has experimentally or computationally demonstrated recursive splice sites at the 3' end of exons. We not only identify these bioinformatically, we demonstrate experimentally as well. In addition, we show that not only can strong SS function as spurious splice substrates, but unexpected so can very weak SS; those that would never be considered a SS.

2 -pp.3-5. The Introduction is overall unclear, muddled, and confusing. The last paragraph is clear, but otherwise the Introduction does not sufficiently represent the history of the EJC in splicing.

We rewrote the introduction to give more attention to the history and connection of EJC to splicing.

3 -p.3. "Cryo-EM structures of prespliceosomal complexes show that U1 snRNA establishes base contacts across the -2 to +6 position for a typical 5'SS…". This is a rather bizarre statement. It is true, but it seems to assert that U1-5'SS base pairing was discovered by cryo-EM, rather than proven by biochemistry and genetics over 30 years ago.

We acknowledge that we were awkward with this, and thank the referee for pointing this out. We meant to highlight that this was directly visualized by cryo-EM structures, but of course this builds on a long history of molecular and genetic experiments. We have revised the text to provide better homage to the history of these studies.

4 -Fig. 1A. This schematic doesn't make much sense at first. It is not clear that the blue/black represents the canonical WT splicing isoform, and the labeled splice sites and red lines represent only cryptic sites and splicing.

We modified the schematic in Figure 1A and figure legend to enhance clarity. We used black font and solid lines to reflect canonical splice sites and splicing. For contrast, we used red colored fonts and dashed lines to signify usage of cryptic splice sites that lie within canonical exons or introns.

5 -Fig. 1C, and 2C,E,F. What exactly are these alternative isoforms? There should be Sanger sequencing information of these PCR products provided.

We thank the reviewer for raising this issue. In Fig 1C and Fig 2, rtPCR of endogenous *unkempt (unk)* and *CG7408* yields multiple amplicons – even in control lanes. These amplicons are obtained from annotated alternative splicing of the same host genes. Our initial submission lacked mention of this. We now provide sashimi plots from RNAseq data to illustrate this point (see updated Figures S1, 2B and S2) and have updated the text that these bands are in fact expected products. In the case of the multiple *CG7408* reporter products, we also confirmed that these correspond to the same set of spliced

products obtained from the endogenous gene. For all products examined, we verified identity using a combination of expected size and sequencing where feasible.

6 -Likewise, what are any of these amplicons supposed to be? The primer binding sites should be shown in a schematic for each gene. Without this information, it is quite difficult to evaluate any of these data.

We have edited the text to reflect that the cryptic splicing events tested were nested within each PCR amplicon. The primer information is provided in Supplementary Table 3 and we have now mapped the onto scaled gene models in Supplementary Figure 2.

7 -1C panel 7 is missing an asterisk for the cryptic product.

We added an asterisk to the bioinformatically predicted spurious product.

8 -All the gels have DNA ladders included, but none are labeled, so the reader doesn't know what size any of the bands are.

Markers have been labeled in the updated figures.

9 -p.7. "spurious exonic 3' SS " and "cluster specifically around exon junctions", etc. The authors need to adopt more precise language. For the RNA molecule in which the cryptic 3'SS is used, it is the 3'SS and it is not exonic. The authors are comparing to a canonical splicing pattern found in WT cells; they need to be more precise to avoid confusion.

We thank the reviewer for pointing out this source of confusion and we agree that splice sites – by definition – lie at the exon/intron boundary. The term "exonic" (3'SS/5'SS) was previously adopted in Boehm et al (*Mol Cell*, 2018), and specifically employs the wildtype/canonical splicing pattern as reference.

We believe that it is vital to communicate that the spurious splice sites examined in this study are 1. found on the exons of wildtype transcripts, near exon-exon junction sequences and 2. are involved in re-splicing. The use of the term "exonic" evokes both of these key ideas. In fact, re-splicing implies use of a splice site from a spliced segment (which is by definition exonic).

We would like to be precise with our language while using terminology that can evoke our key proposals. Therefore, we clearly explain the definition of exonic SS in the earliest reference and emphasize that use of the term "exonic" in relation to wildtype splicing patterns. Thereafter, we continue to use the term exonic 5'SS/3'SS.

10 -p.8. "Our cryptic junction replaces intron 1". How can a junction replace an intron?

We have modified the text to correct this error.

11 -p.8. "this reporter recapitulated normal splicing through activation of annotated 3' SS". To what control is this new reporter compared to show that it recapitulates "normal" splicing? No such control is shown.

We have updated the text to reflect that the genomic *CG7408* reporter yields products that use annotated 3'SS.

12 -p.8. "At face value, this appears consistent with the hypothesis that the EJC regulates splicing of flanking introns." Huh?? This conclusion does not follow from the previous sentences. Perhaps the authors thought they put it elsewhere?

We have reworded this statement. Previous work from the Roignant and Brennecke labs showed that EJC deposition on the *piwi* transcript facilitated removal of neighboring intron 4 (Hayashi G&D 2014, Malone G&D 2014). These studies underscore how recruitment of EJC to exon-exon junctions can influence the splicing of neighboring introns. We were interested in exploring this same concept for *CG7408,* which undergoes EJC-sensitive spurious splicing. Tests using *CG7408* constructs or without introns (genomic and mRNA) yielded canonical products. Our assessment of these results was that the EJC was likely required during pre-mRNA processing, consistent with its requirement for the processing of *piwi.* The current text elaborates on this point.

13 -p.8. Language like "pre-processed exon junctions" and "pre-spliced" is confusing and inaccurate.

We thank the reviewer for their comments. We agree that the terms do not accurately describe our efforts. Instead, we have modified the text to say that these constructs had intron deletions.

14 -p.8 and Figure 2 D, E: There is no evidence here that this is related to EJC deposition. An alternative model, not considered, is that i2 deletion brings an exonic enhancer present in e3 close to the cryptic 3'SS found in e2.

In our original submission, Δi2 and genomic+spacer *CG7408* minigene reporters activated the cryptic exonic 3'SS. These experiments along with controls led us to infer that the EJC deposition masks spurious splice sites. However, we agree with the reviewer's interpretations – it is possible that Δi2 brings an exonic enhancer proximal to the cryptic 3'SS. Here as well, it is possible the EJC may block the activity of the enhancer. Similarly, it is also possible that the spacer enhancers 3'SS activation.

We tested two additional spacer variants (see below 16), which also produced cryptic splicing yielding a total of 4 reporter variants that activate the same spurious splice site. All three spacer sequences are from commonly epitope tags (FLAG, MYC and HA), which do not exist in the Drosophila genome and are not known to regulate splicing. As the same enhancer sequence is unlikely to be present in all three tags, the data supports the involvement of EJC deposition in masking spurious SS. We acknowledge it is theoretically possible these spacer sequences and the e3 sequence may not be neutral and could have some splice enhancer properties. However, our work shows that shifting cryptic 3'SS upstream of EJC deposition - in a largely sequence-independent fashion - is sufficient to activate the sequence in a construct that contains introns.

15 -Likewise the authors have no evidence for the order of intron removal in CG7408.

We thank the reviewer for their point, but we believe our data strongly indicates out of order intron removal as a quality control mechanism to regulate splicing fidelity for *CG7408*. First, we note that EJC LOF reproducibly induces cryptic splicing (Fig 2C), indicating a requirement for this complex. We then demonstrate, using minigene

reporters, that deletion of intron 2 is sufficient to trigger cryptic splicing. This could be because of loss of EJC masking at E2 or because intron deletion brings an enhancer close to the cryptic 3'SS. Two lines of evidence support the former: first, EJC binds exon junctions in the *CG7408* - we include EJC CLIP from the Ephrussi and Ule labs to highlight this (see below). Second, when the spurious 3'SS is separated from EJC recruitment site using spacers we consistently observe cryptic splicing.

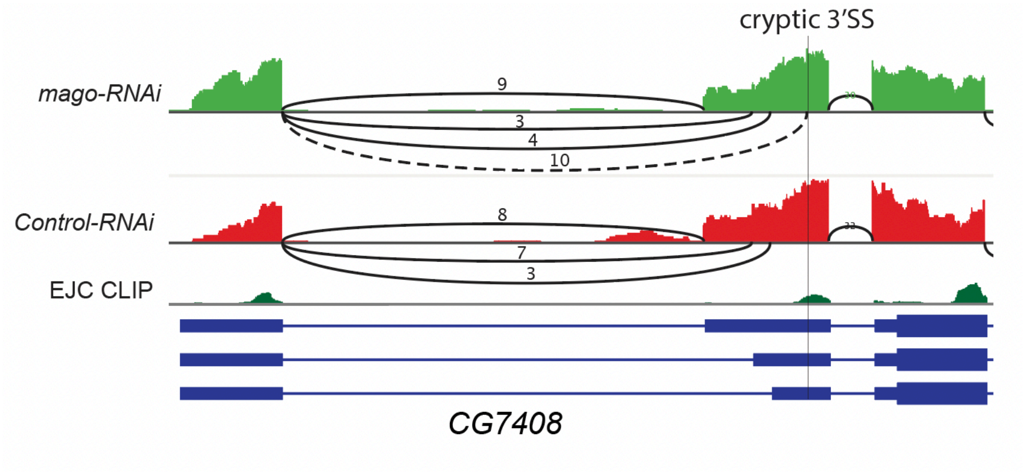

In our view, the aforementioned points strongly support the EJC masking model for suppressing of spurious splice sites – so how does this relate to order of intron removal? In the specific case of *CG7408,* the cryptic 3'SS that marks the end of spurious intron 1 is actually masked by the EJC deposited during the removal of intron 2. Therefore, the EJC-based masking is only relevant in an out-of-order splicing scenario. Thus, as the data indicates EJC masks the spurious 3'SS, it is reasonable to imagine so because of out of order splicing.

To try to provide further evidence of *CG7408* order of intron removal we examined nanopore analysis of co-transcriptional processing (nano-COP) data recently published by the Churchman lab (Drexler, Mol Cell 2020). In this data, nascent RNAs are directly sequenced through nanopores, yielding long read sequences that can show transcript connectivity to understand splicing dynamics. Ghis study utilized nano-COP data to identify 1800 out of order splicing events in Drosophila S2 cells. Therefore, we used these datasets to examine genes with spurious splicing. Unfortunately, we were unable to detect any useful coverage for *CG7408,* which is lowly expressed in S2 cells. However, for two other EJC-suppressed genes studied here (Unkempt, Figure 1C and CKIIß, Figure 4A-C), we observe direct **evidence for out of order splicing** via reads containing a critical upstream intron but with downstream intron removed. Thus, we think there is experimental and nascent processing support for EJC masking of spurious 3'SS. These data were added to Supplementary Figure 4.

16 -Fig. 2F. What is the 36-nt sequence that was added? This is not provided. How do we know that it is a 'neutral' sequence? Could it contain a splicing enhancer? (Honestly, it's not so easy to come up with a neutral sequence.)

The reviewer brings up an astute point, one which we are currently examining. The spacer sequence used in **Figure 2D,F** (genomic + spacer) is a subset of a 3xFLAG tag and we are not aware that this sequence has any splicing modulatory activity.

Nevertheless, given the unusual position of the tag within this construct, it could be a concern. Overall, the activity of the spacer sequence could be related to EJC deposition or splicing modulation, so it is critical to investigate and distinguish between these possibilities.

If the mechanism is based on occlusion, any spacer sequence should suffice, whereas a splicing enhancer would be sequence specific. Therefore, we tested two additional spacer sequences. These sequences will be obtained from other common tags such as HA and MYC (see Figure below). Aberrant products for all three constructs with spacers will provide strong support for a mechanism in which EJC binds and occludes SS at exon-exon junctions.

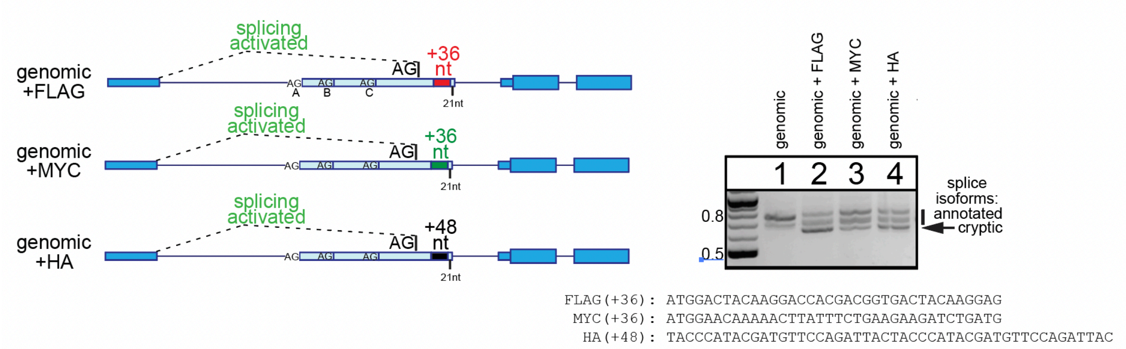

Indeed, in support of our hypothesis, all three spacer sequences show significant activation of the cryptic 3'SS, demonstrated by the production of the aberrant product. Therefore, while we cannot exclude the possibility that the spacers may provide splicing enhancement, our data also strongly indicates that the EJC suppresses activation of cryptic 3'SS at exon junctions by occluding their recognition by the spliceosome. The new data are provided in Figure 2.

17 -p.9 and Fig 2A and 3A. "clear preference in the vicinity of exon junctions but distribution across a wide range of strengths." What is the X-axis? The vast majority of exons are not 500 nt long. Most are ~100-150 nts, so this schematic just shows that the cryptic 5'SS are found within a distance of a typical exon from the WT 5'SS. It looks like the authors used -500 and +500 sequence from the selected WT exon junction, but these are not necessarily all exonic sequence; however, it is not at all clear what sequence the authors use here.

We thank the reviewer for their careful consideration. The distance metric in Figure 2A and 3A represents the minimum distance to a wildtype exon end (see below).

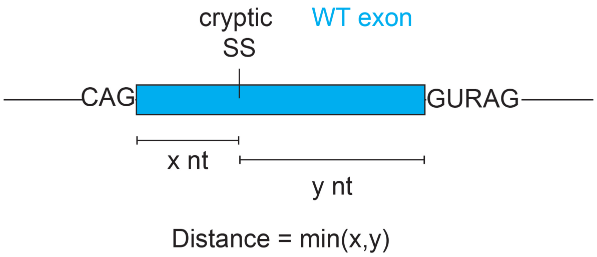

With this configuration, we noticed that the vast majority of spurious SS could be found at < 50 nt from nearest wildtype exon 5'/3'SS (see vertical dashed lines). In addition, we also found SS that were far away from exon ends, but these were few in comparison.

Our goal was to represent the distance between spurious splice sites and the nearest canonical SS (the raw values are plotted), irrespective of exon length. We emphasize that exon size is not normalized in any way and is not meant to suggest anything about average exon lengths in the fly. With regards to our choice of x-limits shown in the metagene (-500, 500), we aimed to select a measure of central tendency that would allow us to include as many spurious SS as possible – the average length of exons with cryptic 3'SS is, in fact, 610 nt.

In Drosophila, intron lengths tend to be quite short (on average ~60nt) and splicing occurs predominantly via intron definition. Hence, while the modal length of exons from spliced genes is ~150 nt, the average exon length is 510 nt. This includes 30% of all unique exons with lengths greater than 500nt and 13% of all unique exons with lengths greater than 1 kb.
The conclusion that would like to draw readers' attention to is that most of the spurious SS enrich within 50 nt from host exon-exon junction, which is proximal to the EJC deposition site.

18 -There are logical inconsistencies. For example, on p.15 the authors correctly admit the they cannot distinguish recursive splicing from alternative splicing; yet, in the next sentence state that they "readily detect re-splicing on all cDNA constructs tested".

We thank the reviewer for pointing out text that is unclear. We have reworded our statements to reflect that we cannot distinguish recursive splicing from alternative splicing "**without further experimentation"**. However, when we design and carry out the appropriate tests, we can readily detect processing from all cDNA constructs which strongly implies that the EJC prevents re-splicing.

19 -p.16. "…our work uncovers an important co-transcriptional function of intron removal…". I do not see any data that address the co-transcriptional nature of the current findings.

We have reworded our statement. Splicing within genes is mostly considered a cotranscriptional process, but we have not conducted any tests to evaluate the stage at which EJC-mediated protection in required.

Typos
20 -p.3. "nucletodies" should be "nucleotides".
21 -p.15. "experimentaion" should be "experimentation".

We thank the reviewer for pointing out these errors. We have corrected the typos.

Reviewer #2: The authors investigate whether the recently reported function of the EJC in suppressing cryptic SS is conserved in fly. They do this by initally performing a reanalysis of previously published EJC component KDs + RNA-seq, finding substantial activation of cryptic SS shared across KDs of the three EJC core proteins. They use RT-PCR to validate a number of these cryptic SS, and then proceed to explore the mechanism of these changes using minigenes for a select few genes. For both 5' and 3' SS they present convincing evidence that the EJC normally blocks recognition of the cryptic SS. They next use similiar minigene experiments to show some proportion of the cryptic events correspond to resplicing, and point out that this is potentially quite common given the canonical SS motifs (i.e. the frequent GT at the end of the exon and the AG at the beginning). They point out that this is concerning for transgene experiments where the processed sequence is introduced since this will not have the benefit of the EJC protecting it from resplicing.

The paper is well written, motivated and clear. It would be helpful to label the exon/intron numbers in all figures to make them easier to connect to the text.

No discussion is given to the strength of the branchpoint sequence for the regenerated introns. It is surprising that such weak (e.g. 0 NNSPLICE scoring) 3' SS are splice-component: is this being compensated by strong BP? If not, what is the authors' explanation for the splicing of such weak SS?

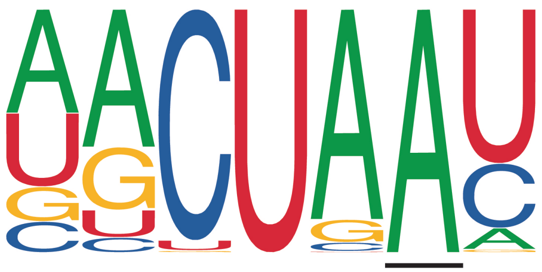

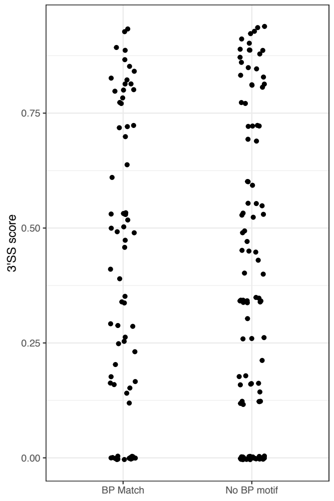

We thank the reviewer for their comments. It is indeed quite surprising that poor splice sites are able to participate in splicing reactions. We intentionally focused on the strength of the splice sites since branchpoint sequences are quite degenerate and challenging to predict. The general view is that the branchpoint adenosine is positionally conserved but the sequence context about this element is less constrained. Nevertheless, we have been cognizant of this element and careful in our experiments to not alter the relationship between BP and 3'SS (In Fig 2F, the spacer sequence does not separate BP and 3'SS).

Encouraged by the reviewer's suggestion, we tried to identify and evaluate the BP sequences for spuriously activated 3'SS. We first derived a consensus BP motif as has been done previously (Pai eLife 2017 and Lim PNAS 2001), by calling motifs within -45 nt and -15 nt from 3'SS using 10000 randomly selected canonical introns. The nucleotide frequency for the top motif is shown on the right and strongly matches those

called in previous studies (Pai eLife 2017); the putative branchpoint in underlined. It is important to emphasize that while this is the top motif, ~50% of introns examined lacked a 75% match. The implication of this statistic is that BPs can be quite diverse.

When we checked spurious 3'SS that lie on exons for this BP motif, we found that 59/118 (50%) splice sites also contained a 75% PWM match. Therefore, it seems that the same proportion of spurious 3'SS and canonical 3'SS have paired upstream BP motifs. We found this to be true irrespective of 3'SS strength as a 3'SS of all strengths are paired with this BP motif. Furthermore, when we compared the scores of 3'SS that had upstream BP motifs versus those that did not, we did not observe gross difference between the two sets. We have added these data to Supplementary Figure 3.

Overall, as splicing reactions require BP adenosines, all spurious 3'SS, irrespective of strength must be paired with a BP adenosine. Our analysis indicates that 50% of regenerated introns may have a motif that resembles such a sequence. We suspect that the activation of these sites in general may be enhanced by the presence of splicing regulatory sequences.

Minor: I'm confused about the statement that "spurious exonic 3' SS. These represent a majority...". From fig 1 it looks like exonic 5' SS are the biggest category?

We have reworded our text to correct this confusion.

Overall this is a nice piece of work with sensible bioinformatic analysis, convincing experiments, and important findings for both splicing biology and functional genetics more broadly.

We thank the reviewer for the positive feedback.

Have all data underlying the figures and results presented in the manuscript been provided?
Large-scale datasets should be made available via a public repository as described in the PLOS Genetics data availability policy, and numerical data that underlies graphs or summary statistics should be provided in spreadsheet form as supporting information.
Reviewer #1: Yes
Reviewer #2: None

PLOS authors have the option to publish the peer review history of their article (what does this mean?). If published, this will include your full peer review and any attached files.

If you choose "no", your identity will remain anonymous but your review may still be made public.

Do you want your identity to be public for this peer review? *For information about this choice, including consent withdrawal, please see our Privacy Policy.*
Reviewer #1: No
Reviewer #2: No

In compliance with data protection regulations, you may request that we remove your personal registration details at any time. (Remove my information/details). Please contact the publication office if you have any questions.

**** Only open attachments or links from trusted senders. Report phishing to infosec@mskcc.org ****
