# Supplementary material for: The Exon Junction Complex and intron removal prevent re-splicing of mRNA
Source: PLoS Genet. 2021 May 25;17(5):e1009563. doi: 10.1371/journal.pgen.1009563 (PMC8184009; doi:10.1371/journal.pgen.1009563)
Supplement: Attachment — Submitted filename: 2021_0410_EJC_PlosGen_2ndreviews for submit.pdf [file pgen.1009563.s013.pdf]

CC: john.greally@einsteinmed.org, teresa.bowman@einsteinmed.org

Dear Dr Lai,

Thank you very much for submitting your Research Article entitled 'The Exon Junction Complex and intron removal prevent re-splicing of mRNA' to PLOS Genetics.
The manuscript was fully evaluated at the editorial level and by independent peer reviewers. The reviewers appreciated the attention to an important topic but identified some concerns that we ask you address in a revised manuscript. The comments are mostly to improve the description in the text and in the figures as to the identity of the tested amplicons. This point is important to address to ensure the ability of future interested scientists to reproduce the work.

We therefore ask you to modify the manuscript according to the review recommendations. Your revisions should address the specific points made by reviewer #1. The revised work can be assessed at the editorial level to help expedite a final decision.

In addition we ask that you:
1) Provide a detailed list of your responses to the review comments and a description of the changes you have made in the manuscript.
2) Upload a Striking Image with a corresponding caption to accompany your manuscript if one is available (either a new image or an existing one from within your manuscript). If this image is judged to be suitable, it may be featured on our website. Images should ideally be high resolution, eye-catching, single panel square images. For examples, please browse our archive. If your image is from someone other than yourself, please ensure that the artist has read and agreed to the terms and conditions of the Creative Commons Attribution License. Note: we cannot publish copyrighted images.

We hope to receive your revised manuscript within the next 30 days. If you anticipate any delay in its return, we would ask you to let us know the expected resubmission date by email to plosgenetics@plos.org.

If present, accompanying reviewer attachments should be included with this email; please notify the journal office if any appear to be missing. They will also be available for download from the link below. You can use this link to log into the system when you are ready to submit a revised version, having first consulted our Submission Checklist.

While revising your submission, please upload your figure files to the Preflight Analysis and Conversion Engine (PACE) digital diagnostic tool. PACE helps ensure that figures meet PLOS requirements. To use PACE, you must first register as a user. Then, login and navigate to the UPLOAD tab, where you will find detailed instructions on how to use the tool. If you encounter any issues or have any questions when using PACE, please email us at figures@plos.org.

Please be aware that our data availability policy requires that all numerical data underlying graphs or summary statistics are included with the submission, and you will need to provide this upon resubmission if not already present. In addition, we do not permit the inclusion of phrases such as "data not shown" or "unpublished results" in manuscripts. All points should be backed up by data provided with the submission. To enhance the reproducibility of your results, we recommend that you deposit your laboratory protocols in protocols.io, where a protocol can be assigned its own identifier (DOI) such that it can be cited independently in the future. Additionally, PLOS ONE offers an option to publish peer-reviewed clinical study protocols. Read more information on sharing protocols at https://plos.org/protocols?utm_medium=editorial-email&utm_source=authorletters&utm_campaign=protocols
Please review your reference list to ensure that it is complete and correct. If you have cited papers that have been retracted, please include the rationale for doing so in the manuscript text, or remove these references and replace them with relevant current references. Any changes to the reference list should be mentioned in the rebuttal letter that accompanies your revised manuscript. If you need to cite a retracted article, indicate the article's retracted status in the References list and also include a citation and full reference for the retraction notice.

PLOS has incorporated Similarity Check, powered by iThenticate, into its journal-wide submission system in order to screen submitted content for originality before publication. Each PLOS journal undertakes screening on a proportion of submitted articles. You will be contacted if needed following the screening process.

To resubmit, you will need to go to the link below and 'Revise Submission' in the 'Submissions Needing

Revision' folder.

Please let us know if you have any questions while making these revisions.

Yours sincerely,

Teresa Bowman
Guest Editor
PLOS Genetics

John Greally
Section Editor: Epigenetics
PLOS Genetics

Reviewer's Responses to Questions
Comments to the Authors:
Please note here if the review is uploaded as an attachment.
Reviewer #1: Overall, this version is much improved. In particular, the introduction is much more clear, coherent, and complete. The results also are better described and the language more clear. However, a few aspects still need clarification.

We thank the referee for their endorsement of the revised work.

1. Figure 1. I appreciate the improved clarity of the model and the addition of the size markers in 1C. However, as far as I can see there are still no indicators as to what the amplicons in the gels are. For example, icons depicting the amplicons like those used in Figure 4D and F would be helpful – except please include an indication of what exons are represented.

To enhance the clarity of the model, we added new genome browser screenshots so that both cryptic 3'SS and cryptic 5'SS examples are shown. We also add a schematic figure of representative mRNA deletion products look like when exonic 5'SS or 3'SS or engaged, and the resulting interpretation of the rt-PCR tests, which in this and all figures will show that shorter mRNA products are observed when cryptic exonic splicing is activated. Hopefully this clarifies this assay which is used in all figures.

The locations of the primers and amplicons of the respective gene models tested in Figure 1 were added as Supplementary Figure 2. The primer sequences used for rt-PCR are listed in Supplementary Table 3.

2. This also relates to Question 5, which was "what exactly are the amplicons?" The authors say that they confirmed amplicons by Sanger sequencing or by size, and I'm mostly ok with that (although they do not tell us which amplicons were confirmed by sequencing), but they don't tell us what the amplicons actually are. Without information as to the identity of the amplicons, this work could never be replicated.

The amplicons are shown in genome schematics in Supplementary Figure 2, and all the rt-PCR primer sequences are provided in Supplementary Table 3. It will be straightforward for anyone to replicate these tests, which we have done ourselves and we know all of the validated examples to provide robust changes. Moreover, the basis of our study was analysis of public RNA-seq data, which anyone can download, and refer directly to any of the cryptic junctions we have annotated in Supplementary Table 1.

3. In Figure 4F, the amplicon icons on the right seem too high, i.e. they aren't aligned with the gel bands.

We have adjusted the figure to align the icons better.

4. Question 19. The authors say that they re-worded their statement about "...our work uncovers an important co-transcriptional function of intron removal...". I maintain that their work does not address the co-transcriptionality of the current findings – and the authors say that they agree. However, on page 19 in the last sentence of the Discussion, they still say "our work uncovers an important co-transcriptional function of intron removal".

The referee has raised this point before and we softened relevant restatements in response. However, we must highlight what is a substantial achievement of our work regarding the importance of out-of-order splicing. Others (as cited in the text) have shown that out-or-order splicing can be detected, and we extend

these observations here including with data from long-read nascent co-transcriptional processing data (thus addressing detection of co-transcriptionality). But, very few studies have shown that out-of-order splicing is necessary for proper mRNA processing, as opposed to occurring but being incidental and dispensable for proper mRNA maturation. As we have shown, out-of-order splicing can be requisite to prevent cryptic splicing, by appropriate recruitment of the EJC.

We don't want to debate this point further so we are removing "co-transcriptional" from page 19, but it is unfortunate not to have an interactive discussion of this topic.

5. Figure 5 C. Again, it would really help the reader to have explicit schematic or icon next to the gels that show what the amplicons are. It is also unclear why the bands using the transgenic vs genetic specific are so different in size.

The referee has commented on this previously, and we do not feel it is informative by this figure in the paper. The paper is about cryptic splicing that removes content from the normal mature mRNA products, so the cryptic isoforms are always shorter than wildtype. However, to ensure clarity when the assay is first introduced, we inserted schematic panels into Figure 1 that illustrate that either 5'SS and 3'SS cryptic exonic splicing will result in shorter mRNA products bearing internal deletions relative to the normal product.

Regarding the comment "*It is also unclear why the bands using the transgenic vs genetic specific are so different in size.*" It appears the referee has not thought about the experiment carefully. If the bands in transgenic vs endogenous situation were the same, then how could we conclude that we had assayed the transgene as opposed to endogenous transcripts?

Instead, the transgenic tests use a gene-specific primer and a vector primer, designed specifically to generate band sizes that are very different from endogenous, so that we could tell we have amplified the transgene.

Seeing how this experimental logic may not be clear by some readers, we have adjusted the figure legend to point this out, although it was already stated in the existing figure legend that transgene-specific primers were used.

6. On p. 34, Figure 5 Legend is labeled incorrectly. There are 2 legends labeled "Figure 4".

We have corrected this error. Thanks for pointing it out.

Reviewer #2: I was already positive about this manuscript and the authors have improved it on revision. I appreciate them also having taken the time to look into whether BP sequence might account for some of the variation they see. The other reviewer was more critical so I will leave it to them to determine whether their concerns are addressed - it certainly appears the authors have made substantial efforts to do so.

We thank the referee for their strong endorsement of our manuscript.

Have all data underlying the figures and results presented in the manuscript been provided?
Large-scale datasets should be made available via a public repository as described in the PLOS Genetics data availability policy, and numerical data that underlies graphs or summary statistics should be provided in spreadsheet form as supporting information.
Reviewer #1: None
Reviewer #2: None

PLOS authors have the option to publish the peer review history of their article (what does this mean?). If published, this will include your full peer review and any attached files.

If you choose "no", your identity will remain anonymous but your review may still be made public.

Do you want your identity to be public for this peer review? *For information about this choice, including consent withdrawal, please see our Privacy Policy.*
Reviewer #1: No
Reviewer #2: No
